# High-Quality Assemblies for Three Invasive Social Wasps from the *Vespula* Genus

**DOI:** 10.1534/g3.120.401579

**Published:** 2020-08-28

**Authors:** Thomas W. R. Harrop, Joseph Guhlin, Gemma M. McLaughlin, Elizabeth Permina, Peter Stockwell, Josh Gilligan, Marissa F. Le Lec, Monica A. M. Gruber, Oliver Quinn, Mackenzie Lovegrove, Elizabeth J. Duncan, Emily J. Remnant, Jens Van Eeckhoven, Brittany Graham, Rosemary A. Knapp, Kyle W. Langford, Zev Kronenberg, Maximilian O. Press, Stephen M. Eacker, Erin E. Wilson-Rankin, Jessica Purcell, Philip J. Lester, Peter K. Dearden

**Affiliations:** * Genomics Aotearoa and Department of Biochemistry, University of Otago, Dunedin 9054, Aotearoa, New Zealand; † Pathology Department, University of Otago, Dunedin, Aotearoa, New Zealand; ‡ Centre for Biodiversity and Restoration Ecology, School of Biological Sciences, Victoria University of Wellington, PO Box 600, New Zealand; § School of Biology, Faculty of Biological Sciences, University of Leeds, LS2 9JT, United Kingdom; ** School of Life and Environmental Sciences, University of Sydney, NSW 2006, Australia; †† Phase Genomics Inc., 1617 8th Ave N, Seattle, WA 98109; ‡‡ Department of Entomology, University of California, Riverside, CA 92521

**Keywords:** Vespula germanica, Vespula pensylvanica, Vespula vulgaris, Hymenoptera, social insects, genomes

## Abstract

Social wasps of the genus *Vespula* have spread to nearly all landmasses worldwide and have become significant pests in their introduced ranges, affecting economies and biodiversity. Comprehensive genome assemblies and annotations for these species are required to develop the next generation of control strategies and monitor existing chemical control. We sequenced and annotated the genomes of the common wasp (*Vespula vulgaris*), German wasp (*Vespula germanica*), and the western yellowjacket (*Vespula pensylvanica*). Our chromosome-level *Vespula* assemblies each contain 176–179 Mb of total sequence assembled into 25 scaffolds, with 10–200 unanchored scaffolds, and 16,566–18,948 genes. We annotated gene sets relevant to the applied management of invasive wasp populations, including genes associated with spermatogenesis and development, pesticide resistance, olfactory receptors, immunity and venom. These genomes provide evidence for active DNA methylation in Vespidae and tandem duplications of venom genes. Our genomic resources will contribute to the development of next-generation control strategies, and monitoring potential resistance to chemical control.

Social wasps (Hymenoptera: Vespidae) are remarkable because their highly eusocial lifestyle appears to have evolved independently of other eusocial Hymenoptera (Piekarski *et al.* 2018; Hines *et al.* 2007; Peters *et al.* 2017). The eusocial lifestyle is characterized by overlapping generations of adults living together, cooperative care of offspring and reproductive division of labor (Michener and Brothers 1974). Along with their foraging flexibility and predatory ability, eusociality appears to play a major role in the ecological success of social waps (Grimaldi and Engel 2005; Lester and Beggs 2019).

Vespid wasps can be effective pollinators of plants including ivy or orchid species (Cheng *et al.* 2009; Jacobs *et al.* 2010). They are generalist predators and effect biological control of some pest species (Donovan 2003). Their ecological success can also be problematic. Invasive colonies of common wasps (*Vespula vulgaris*) can contain up to 230,000 workers, while nests of the western yellowjacket (*Vespula pensylvanica*) containing up to half a million individuals have been observed (Lester and Beggs 2019). Colonies are smaller in the native ranges (Archer and Turner 2014 p. 61). In New Zealand’s native beech forests, Vespid wasp populations can reach up to 40 nests per hectare and have a biomass similar to, or greater than, the combined biomasses of birds and mammals (Thomas *et al.* 1990; Lester *et al.* 2017). These invasive Vespid populations (Figure 1; Lester and Beggs 2019) have major impacts on ecosystems because of their large colony sizes, reproductive capacity and flexible predation.

**Figure 1:**
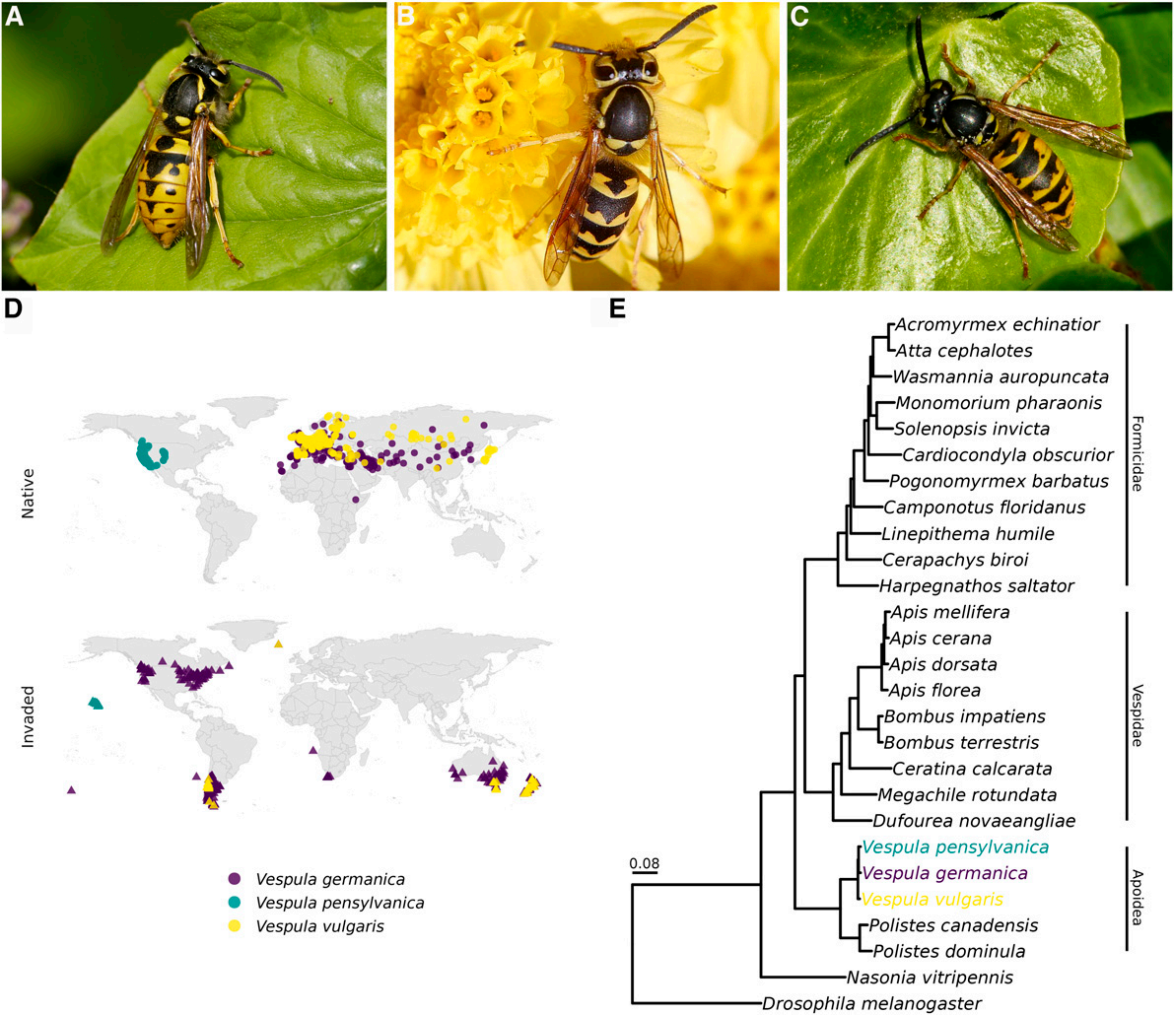
Vespula species are major invasive pests. A–C. Examples of (A) *Vespula germanica*, (B) *Vespula pensylvanica* (Jon Sullivan, public domain), and (C) *Vespula vulgaris* (Sid Mosdell, CC BY 2.0). D. Native and invaded ranges of the sequenced Vespula species. E. Rooted phylogenetic species tree of *Vespula* species and other sequenced Hymenoptera, with *Drosophila melanogaster* as an outspecies, derived from 415 orthogroups using OrthoFinder.

Current control methods for vespid wasps are limited, with pesticides containing Fipronil being the most common and widespread chemical control method (Edwards *et al.* 2017). The use of this neurotoxic pesticide over large areas, and over consecutive years, may select for resistance. Fipronil resistance has been observed in other economically important insect pests (Matsumura *et al.* 2008; reviewed in Feyereisen *et al.* 2015). Next-generation pest control technologies, including gene drives, have been proposed as part of an alternative solution for controlling or eradicating invasive social wasps (Dearden *et al.* 2018). Targets for genetic modification include developmental genes associated with wasp fitness or fecundity (Lester and Beggs 2019; Dearden *et al.* 2018). Gene drives that have immune system targets have been proposed and developed in the laboratory for other pests such as mosquitoes (*e.g.*, Gantz *et al.* 2015; Dong *et al.* 2018). Detailed knowledge of *Vespula* genomes is required to ensure that targeted control of Vespids does not affect other beneficial Hymenoptera, such as honeybees or biocontrol agents.

Five genomes from the *Polistes* genus of the Vespidae subclade are available in the NCBI Genome database. Genomes for *Polistes canadensis* and *Polistes dominula* were assembled from short reads (Standage *et al.* 2016; Patalano *et al.* 2015). Genomes for *Polistes dorsalis*, *Polistes fuscatus* and *Polistes metricus*, based on Pacific Biosciences single-molecule sequencing, were recently deposited in the NCBI database (Miller *et al.* 2020). The three long-read genomes have improved contiguity compared to the short-read genomes produced for *P. dominula* and *P. canadensis* (Standage *et al.* 2016; Patalano *et al.* 2015; Miller *et al.* 2020). Here, we report chromosome-level assemblies for three *Vespula* species that are invasive pests in their introduced ranges (Figure 1; Lester and Beggs 2019). We undertake manual and computational annotation and phylogenomic analyses, with emphasis on sets of genes relevant to chemical control, olfaction, venom and DNA methylation. These resources will be useful for understanding the biology of vespid wasps, developing next-generation control strategies, and monitoring resistance to current chemical control.

## Materials and Methods

### Genome assembly and scaffolding

*Vespula pensylvanica* samples were collected in Volcano, Hawaii in August 2017 (lat 19.43, lon -155.21). The *V. pensylvanica* assembly was produced by Dovetail Genomics (https://dovetailgenomics.com/), starting with an Illumina library generated from a single haploid male to produce 115.5 Gb of 150bp paired-end reads. After trimming adaptors and removing bases or truncating reads with low quality scores, the draft genome was assembled with Meraculous 2.2.6 using a kmer size of 55, which produced the best fit with a constrained heterozygous model (Chapman *et al.* 2011, 2016). Chromosome-scale scaffolds were generated using Chicago and Dovetail Hi-C data from diploid females, and implemented using Dovetail’s proprietary HiRise software.

We collected *V. vulgaris* samples in May 2015, from Richmond Hill Forest Park, New Zealand (lat -41.3292, lon 173.4637). *V. germanica* samples were collected in March 2018, from Lincoln, New Zealand (lat -43.6384, lon 172.4784). We used libraries generated from a single, haploid male to produce 17.1 Gb of 150b paired-end reads and 27.3 Gb of 125b paired-end reads, respectively. After trimming adaptor sequences, removing contaminants and verifying pairing with BBMap 38.00 (Bushnell 2014), we assembled draft genomes with Meraculous 2.2.6 (Chapman *et al.* 2011, 2016). We repeated assembly with a range of parameters, and used BUSCO analysis (Simão *et al.* 2015), assembly size and contiguity statistics to choose the best set of parameters for each dataset. Code for running and assessing the assemblies is hosted at https://github.com/tomharrop/vger-illumina and https://github.com/tomharrop/vvul-illumina. Draft genomes were scaffolded by Phase Genomics using Hi-C data generated from pools of 20 larvae. Chromatin conformation capture data were generated using Phase Genomics Proximo Hi-C 2.0 Kit, which is a commercially available version of the Hi-C protocol (Lieberman-Aiden *et al.* 2009). The Phase Genomics Proximo Hi-C genome scaffolding platform was used to create chromosome-scale scaffolds from the corrected assembly as described Bickhart *et al.* (2017).

### Genome curation

Command-line arguments and scripts can be found at https://github.com/jguhlin/vespula_paper. Assembled genomes were cleared of contamination by removing contigs which had BLAST taxonomy results which did not include *Polistes*, *Vespula*, or the word “wasp”. Contigs without BLAST results were kept if they contained predicted genes found in a Hymenoptera orthogroup from an initial gene prediction. Remaining contigs were only kept if they fell within 2 standard deviations of mean GC% of our kept contigs. The largest 25 chromosomes of *V. pensylvanica* were renamed to chromosomes and ordered according to size. Our *V. vulgaris* and *V. germanica* assemblies were aligned to the *V. pensylvanica* genome using D-GENIES, which inserts contigs into syntenic locations, flanked by 100 *N*s, to assign chromosome names to those most related to those in *V. pensylvanica* (Cabanettes and Klopp 2018). Scaffolds were numbered with four digits in order of size.

### Repeat masking

Repeats were identified using RepeatModeler 2.0.1 (github.com/Dfam-consortium/RepeatModeler) and RepeatMasker 4.0.9 (repeatmasker.org/RMDownload.html) via the funannotate pipeline (Palmer and Stajich 2019).

### RNA sequencing

*Vespula vulgaris* queens, workers, and larvae were sampled from mature nests in the native range of Belgium and the introduced range in New Zealand and total RNA transcriptome data were generated as described by Gruber *et al.* (2019).

### Gene prediction

We performed iterative gene prediction using the Funannotate pipeline v1.6.0, manual annotation, and extrinsic protein evidence (Palmer and Stajich 2019). For *V. vulgaris* we used RNA-seq reads (described in the previous section) from *V. vulgaris* queens, workers, and larvae as additional evidence. The reads were trimmed with sickle (github.com/najoshi/sickle) and aligned to our assembly using STAR in two-pass mode (Dobin *et al.* 2012). Gene predictions were performed on the assembly using funannotate predict with the RNA alignments and extrinsic protein of all *Vespula* proteins from NCBI, *Apis mellifera*, *Nasonia vitripennis*, and the UniProt SWISS-PROT database (Boutet *et al.* 2007; Pruitt *et al.* 2005; The Honeybee Genome Sequencing Consortium 2006; Werren *et al.* 2010).

Initial predictions were evaluated with GeneValidator with UniProt SWISS-PROT database as the high-quality targets. Genes whose protein predictions scored > 90 were used to train Augustus via the optimize_augustus.pl script (Drăgan *et al.* 2016). The prediction step of funannotate was then re-run as before using the trained vvulg AUGUSTUS species definition. This allowed retention of high-quality gene predictions from the target species to be used as a training set for AUGUSTUS gene prediction, a component of the funannotate pipeline. This process was repeated for *V. pensylvanica* and *V. germanica* using *V. vulgaris* species definition as the initial AUGUSTUS species in the first iteration of gene prediction, generating a species-specific configuration in the following round. This two-step gene prediction with validation and training using high-confidence gene calls between the first and second round allowed for the creation of species-specific AUGUSTUS models.

### Manual curation

Genes were manually curated in WebApollo (Lee *et al.* 2013). These manual annotations took precedence over intersecting computational gene predictions. Manual annotation was performed on *V. vulgaris* and lifted over to *V. germanica* and *V. pensylvanica* where possible.

### Gene family specific predictions

Gene-family specific predictions were enhanced using AUGUSTUS-PPX for the LGIC and Olfactory families (Keller *et al.* 2011). Protein sequences of interest from external sources were clustered based on bitscore using BLAST+ and MCL (Dongen 2000). Clusters of protein sequences were converted to protein profiles via AUGUSTUS tool msa2prfl.pl Assemblies were searched with fastBlockSearch and gene prediction was performed on matched regions with an additional flanking sequence of 1kbp. These predictions took precedence over intersecting computationally predicted genes.

### Annotation

Further annotation was performed with funannotate using InterProScan 5.32-71.0 (Mitchell *et al.* 2019). Genes were renamed using custom scripts. Protein predictions were compared with GeneValidator to both our Hymenoptera + *Drosophila* Protein Set and UniProt-SwissProt to generate GV scores and statistics. Proteins were compared with the publicly available genomes from Hymenoptera base using OrthoFinder (Elsik *et al.* 2016; Emms and Kelly 2019).

### Methylation analysis

Nucleotide and dinucleotide content of gene body sequences were calculated using a custom perl script. CpG[o/e] was calculated as the number of CpG dinucleotides divided by the number of C nucleotides times the number of G nucleotides. The number of components in CpG[o/e] distributions was estimated in R (R Core Team 2015) using mclust model-based clustering (Scrucca *et al.* 2016). The best fitting model was identified among several non-nested models using Bayesian information criteria (BIC).

### Data availability

Raw sequence data are hosted in the NCBI Sequence Read Archive under accession PRJNA643352. Assembled genomes are available on GenBank under accessions JACSDY000000000 (*Vespula pensylvanica*), JACSDZ000000000 (*Vespula germanica*) and JACSEA000000000 (*Vespula vulgaris*). Supplemental material available at figshare: https://doi.org/10.25387/g3.12885599.

## Results and discussion

### Genome assemblies and annotation

**Figure 2:**
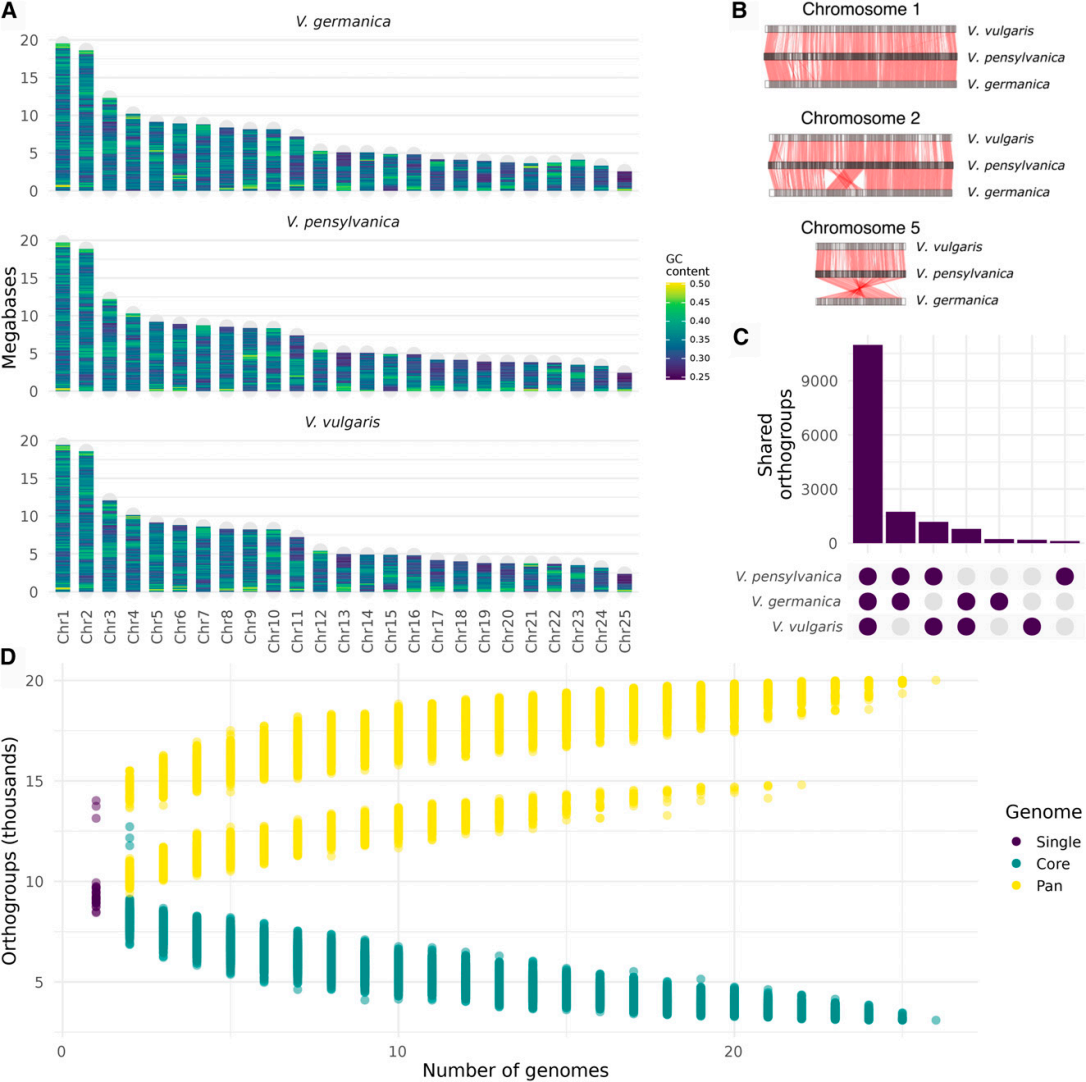
A. Assembled chromosome lengths of the three *Vespula* species. Chromosomes were named in order of scaffold length in *V. pensylvanica*. B. Synteny between selected chromosomes of *Vespula* species. Chromosome 1 has minor translocations and inversions but is syntenic overall. Chromosome 2 has a larger inversion between *V. pensylvanica* and *V. germanica* that is not present in V. vulgaris. Chromosome 5 has a large translocation and inversion between *V. pensylvanica* and *V. germanica*. Chromosome lengths are scaled to chromosome 1. C. Shared orthogroups between the three *Vespula* species. D. The pan and core genomes of Hymenoptera. The core genome (the set of orthogroups present in all genomes sampled) decreases as more genomes are sampled, to a final core genome size of 3,092 orthogroups when all 26 hymenopteran genomes are compared. The pan genome (the total set of orthogroups present in one or more of the sampled genomes) does not continue to grow as more genomes are sampled, indicating a closed hymenopteran pan genome. We predicted more orthogroups (13,141–14,022 orthogroups) in *Vespula* species than in non-*Vespula* Hymenoptera (median 9,193 orthogroups), which resulted in a larger pan genome in comparisons that included *Vespula* species. We only analyzed genes that were assigned to an orthogroup, and we plotted a random subset of 1,000 comparisons for genome numbers that resulted in more than 1000 comparisons.

We used a combination of short-read Illumina sequencing and Hi-C scaffolding to assemble draft genomes for *V. germanica*, *V. pensylvanica*, and *V. vulgaris*. The genomes each contain 176–179 Mb of total sequence assembled into 25 superscaffolds (Figure 2A; Supplementary table 1; N_50_ length 8.30–8.53 Mb), which likely represent the 25 chromosomes observed in *Vespula* karyotypes (Hoshiba *et al.* 1989). Each draft genome also contains 10–200 unanchored scaffolds (N_50_ lengths 1.77–2.28 kb; Supplementary table 2). These genomes are similar in size to the genomes of the closely related European paper wasp, *Polistes dominula* (Standage *et al.* 2016), and the red paper wasp, *Polistes canadensis* (Patalano *et al.* 2015). However, the contiguity in our *Vespula* assemblies is higher than Illumina-based assemblies of other Vespidae, and comparable to the latest-generation *Apis mellifera* assembly (Table 1). We ordered and named scaffolds in the *Vespula* assemblies based on scaffold length in *V. pensylvanica*. The three genomes are highly syntenic, with evidence of some structural rearrangements (Figure 2B; Supplementary Figure 1). Repeat masking masked 17.86%, 18.75% and 18.71% of the *V. vulgaris*, *V. pensylvanica*, and *V. germanica* genomes, respectively. We predicted 16,751, 17,854, and 19,142 genes for *V. vulgaris*, *V. germanica*, and *V. pensylvanica*, respectively. We found between 92.2% and 96.0% of expected single-copy orthologs using BUSCO with the Hymenoptera lineage dataset (Simão *et al.* 2015). The contiguity of our *Vespula* assemblies and completeness of our annotations indicates that the combination of short-read sequencing and Hi-C scaffolding on haploid material is an effective strategy for assembling high-quality hymenopteran genomes.

**Table 1:** Comparison of Vespidae and honeybee genome assembles. 1. Apart from our Vespula assemblies, repeat content was counted from lowercase nucleotides in published assemblies

Species	Sequencing Strategy	Total Sequence	Largest	Scaffolds/ contigs	*N*_50_ length	Ns	Gaps	Repeats^1^ (%)
*Vespula vulgaris*	Illumina + Hi-C	176,275,134	19,426,332	35	8,304,510	4,147,610	49,679	17.12
*Vespula germanica*	Illumina + Hi-C	178,312,246	19,524,135	133	8,396,154	1,783,864	18,963	18.89
*Vespula pensylvanica*	Illumina + Hi-C	179,379,562	19,704,315	225	8,532,720	444,951	4,987	19.15
*Polistes canadensis*	Illumina	211,202,212	3,185,661	3,836	521,566	14,106,256	15,755	41.60
*Polistes dominula*	Illumina	208,026,220	7,126,315	1,483	1,625,592	7,426,626	14,286	44.50
*Polistes fuscatus*	PacBio Sequel + Illumina + Dovetail	219,116,742	19,629,704	187	9,116,088	4,436,170	1,204	41.79
*Polistes metricus*	PacBio Sequel + Illumina	219,838,961	15,979,625	216	4,634,047	1,082,619	459	44.48
*Polistes dorsalis*	10x Genomics	209,288,276	20,305,868	5,129	5,372,633	4,771,923	8,816	42.35
*Apis mellifera* 4.5	Sanger + SOLiD + 454	250,287,000	29,893,408	5,321	13,219,345	21,165,099	12,690	5.28
*Apis mellifera* HAv3.1	PacBio + 10x Chromium + BioNano + Hi-C	225,250,884	27,754,200	177	13,619,445	1,313,614	51	44.63

To predict divergence time of *Vespula* species from *Polistes*, we reconstructed a phylogeny using other hymenopteran genomes. Based on a published estimate for Hymenoptera (Peters *et al.* 2017), the divergence time of Vespid wasps from their last common ancestor with *P. dominula* is estimated to be 51 million years (95% CI: 34–71 million years). Mitochondrial genomes suggest a divergence time of 75 mya (Huang *et al.* 2019). Scaling our ultrametric phylogenetic tree to the former estimate places separation of *V. vulgaris* from *V. pensylvanica* and *V. germanica* at ∼6 mya, and separation of *V. pensylvanica* from *V. germanica* at 4.5 mya (Supplementary Figure 2).

We manually curated 361 gene models in *V. vulgaris* and used these curations to improve automated prediction steps for the other two species. During manual curation, we focused on a range of gene sets relevant to the evolution and applied management of invasive *Vespula* spp., including olfactory receptors, pesticide resistance, immunity and viruses, venom, and spermatogenesis and development.

To investigate relationships between *Vespula* genes, we clustered predicted proteins into orthogroups with predicted proteins from the Hymenoptera Genome Database, using *Drosophila melanogaster* as the outgroup (Hoskins *et al.* 2015; Elsik *et al.* 2016; Emms and Kelly 2019). Each orthogroup contains a set of genes putatively descended from a single gene in the last common ancestor of the species represented in the orthogroup. Between 82.6% and 88.4% of our predicted *Vespula* proteins belonged to orthogroups. *V. vulgaris* shares 12,560 and 12,084 orthogroups with *V. pensylvanica* and *V. germanica*, respectively, and *V. pensylvanica* shares 13,209 orthogroups with *V. germanica* (Figure 2C). Orthogroups including other hymenopteran species allowed us to predict the core- and pan-genomes for Hymenoptera (Figure 2D). This analysis suggests that Hymenoptera have a closed pan-genome, because as we include more genomes the rate of discovery of new orthogroups decreases. We also observed more orthogroups in our Vespid genomes than in other Hymenoptera, which could indicate over-prediction resulting from our annotation.

93.7–94.5% of predicted orthogroups in *P. canadensis* and 96.0–96.9% in *P. dominula* clustered into orthogroups that contained a *Vespula* gene. *Vespula* genes from our annotations were present in 58.9–62.8% of all orthogroups across Hymenoptera and *D. melanogaster*, compared to 40.7% and 41.2% for *P. canadensis* and *P. dominula*, respectively (Table 2). Other hymenopteran species had a member in 37.8–44.5% of othogroups, indicating gene families may be missing in predictions among other species. Most of the genes we annotated were in shared orthogroups, with 100 genes (0.1–0.4% per species) in species-specific orthogroups. Other hymenopteran genomes had 0–3.3% (mean 1.7%) of genes in species-specific orthogroups. Our annotation results suggest that Vespid wasps have more genes than other Hymenoptera and/or gene annotation in other Hymenoptera is incomplete. This could be resolved by re-annotation of other hymenopteran genes using a comparative approach.

**Table 2:** Gene content and orthogroup representation for selected hymenopteran genomes

Species	Genes	Orthogroups represented	Orthogroups represented (%)	Species-specific orthogroups	Genes in species-specific orthogroups (%)
*Vespula vulgaris*	16,751	13,141	58.9%	5	0.1%
*Vespula germanica*	17,854	13,739	61.6%	23	0.4%
*Vespula pensylvanica*	19,142	14,022	62.8%	11	0.1%
*Polistes canadensis*	10,518	9,086	40.7%	12	0.2%
*Polistes dominula*	11,069	9,193	41.2%	27	0.5%
*Apis mellifera*	14,064	8,949	40.1%	35	0.6%
*Nasonia vitripennis*	14,647	8,901	39.9%	616	11.1%

### Evidence for active DNA methylation

**Figure 3:**
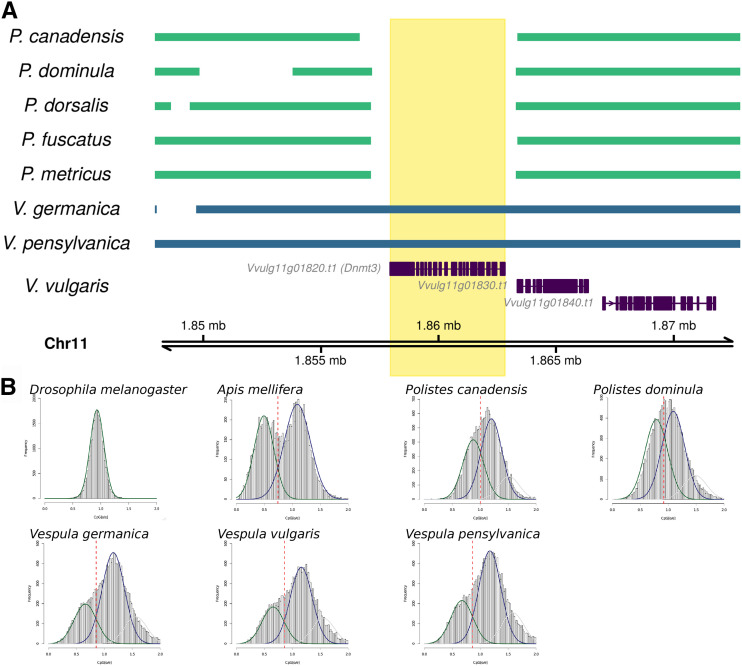
A. Whole-genome alignments of five *Polistes* genomes and genomes of *V. germanica* and *V. pensylvanica* against Chr11 of the V. vulgaris genome, showing the Dnmt3 locus (Vvulg11g01820) and the surrounding 20 kb. None of the *Polistes* assemblies have regions that are homologous to the Dnmt3 locus, and annotations for *P. canadensis* and *P. dominula* do not include a Dnmt3 gene (Standage *et al.* 2016; Patalano *et al.* 2015; Bewick *et al.* 2017). All three *Vespula* genomes encode an ortholog of Dnmt3. B. Frequency histograms of CpG[o/e] observed in coding sequences of *Drosophila melanogaster*, *Apis mellifera*, *Polistes canadensis*, *Polistes dominula*, *Vespula germanica*, *Vespula vulgaris* and *Vespula pensylvanica*. The y-axis depicts the number of genes (Frequency) with CpG[o/e] values given on the x-axis. The distribution of CpG[o/e] in Vespid wasps is a trimodal distribution, with a low-CpG[o/e] peak consistent with the presence of historical DNA methylation in all five Vespid species.

DNA methylation has been functionally linked to caste specification in honeybees and ants and division of labor in honeybees (Herb *et al.* 2012; Kucharski *et al.* 2008; Bonasio *et al.* 2012; Guan *et al.* 2013). DNA methylation may be integral for aspects of eusociality (reviewed by Li-Byarlay *et al.* 2013), although recent studies have found no consistent link (Bewick *et al.* 2017; Glastad *et al.* 2017). In mice, Dnmt3 enzymes catalyze *de novo* DNA methylation (reviewed by Goll and Bestor 2005). The genomes of *P. canadensis* and *P. dominula* do not encode a *Dnmt3* homolog (Standage *et al.* 2016; Patalano *et al.* 2015; Bewick *et al.* 2017). While this manuscript was under revision, three new *Polistes* genomes based on Pacific Biosciences single-molecule sequencing were deposited in the NCBI database (Miller *et al.* 2020). Genome annotations have not been reported for the three long-read *Polistes* genomes. We aligned all five *Polistes* genomes and our *V. germanica* and *V. pensylvanica* genomes against Chr11 of the *V. vulgaris* genome, which encodes *Dnmt3*. None of the five *Polistes* genomes have a region that aligns to the *V. vulgaris Dnmt3* locus (Figure 3A). All three *Vespula* genomes encode an ortholog of *Dnmt3*, indicating the lack of this gene in *Polistes* is due to gene loss following the divergence from the *Vespula* lineage approximately 50 million years ago (Peters *et al.* 2017). *Vespula* genomes all contain a single ortholog of *Dnmt1* and have evidence of active DNA methylation (Figure 3B; Supplementary Figure 3).

### Gene content and duplications

We found more olfactory receptor (OR) genes in *Vespula* genomes than in the genomes of *P. dominula* and *P. canadensis*. We predicted 120 OR genes in *V. vulgaris*, 133 in *V. germanica*, and 102 in *V. pensylvanica*. Annotations for *P. dominula* and *P. canadensis* contain 94 and 72 OR genes, respectively (Supplementary table 3). In contrast, honeybee and *Nasonia* genomes encode more OR genes (170 and 301, respectively; Robertson and Wanner 2006; Robertson *et al.* 2010). *Vespula* OR genes cluster into 28 orthogroups. The co-receptor *Orco* is present in all genomes and forms a stable orthogroup (orthogroup 7148; Larsson *et al.* 2004; Jones *et al.* 2005). In contrast, there are significant expansions of particular OR orthogroups in the Vespid wasps, and these differ from the groups expanded in *Nasonia* and honeybee (Robertson *et al.* 2010; Robertson and Wanner 2006). Orthogroup members are arranged in expanded tandem arrays on chromosome 3 (orthogroup 51), chromosome 13 (orthogroup 2434), and chromosome 25 (orthogroup 232) of *Vespula* genomes. In these clusters, numbers of genes vary between species, implying that duplications and deletions are recent and ongoing. Variation in olfactory receptors between wasp species, and between these wasps and other Hymenoptera may indicate species-specific olfactory biology. These may be key to understanding the social behavior and pheromone signaling systems present within these species.

**Figure 4:**
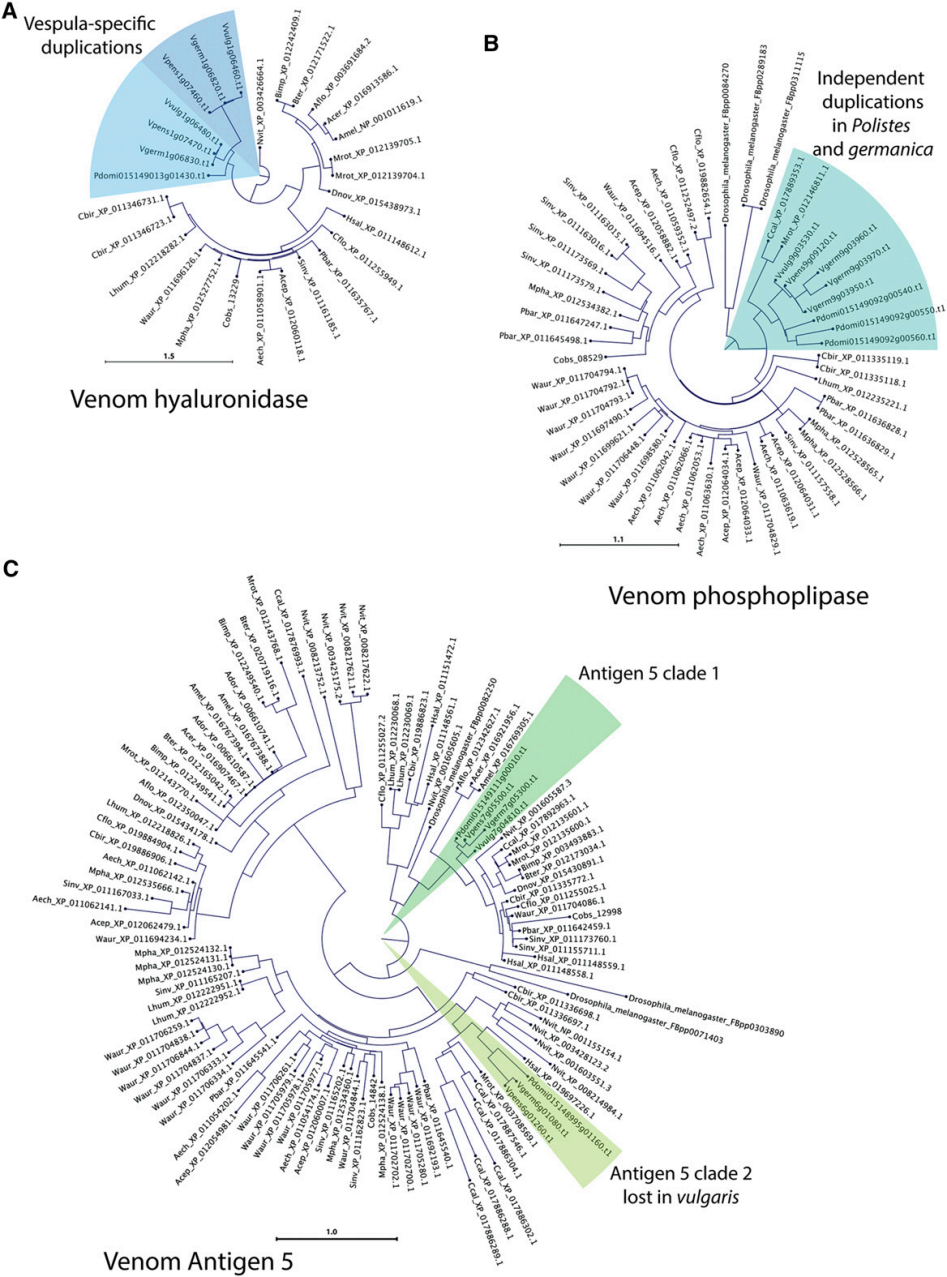
Ultrametric trees of orthogroups encoding Vespid wasp venom components in insect genomes. A. Venom hyaluronidase phylogram showing duplication of this gene in Vespid genomes but not *Polistes*. B. Venom phospholipase phylogram indicating independent duplications in *Polistes* and *V. germanica* from a single gene ancestor in related Hymenoptera. C. Antigen 5 phylogram, indicating two genes encode this venom component in Vespid wasps and *Polisties*, one of which is missing from the *V. vulgaris* genome.

Some of the genes encoding venom components also have variable copy numbers in *Vespula* genomes. The major allergens in *Vespula* venoms are phospholipase A1, hyaluronidase, and antigen 5 (Biló *et al.* 2005; Kolarich *et al.* 2007). Phospholipase A1 is found in three tandem copies in the *P. dominula* and *V. germanica* genomes (chromosome 9 in *Vespula*), and one copy in each of the *V. pensylvanica* and *V. vulgaris* genomes. The phylogenetic placement of these duplicates in *Polistes* and *V. germanica* (Figure 4) implies that these are independent amplifications. The hyluronidase gene is duplicated in our three Vespid genomes, but not *P. dominula*. These are tandem duplications that appear to have been present in the last common ancestor of the three *Vespula* species. *P. dominula*, *V. germanica* and *V. pensylvanica* also have two Antigen 5 genes, but these duplications appear ancient before the common ancestor of Hymenoptera (Figure 4). In *Vespula* species, one copy is on chromosome 6 and one is on chromosome 7. In *V. vulgaris*, the chromosome 6 copy is absent. Duplication of venom genes in Vespids is no surprise, given the importance of venom to their life cycle.

Primary chemical control of *Vespula* populations is through the use of baits containing a low concentration of the phenylpyrazole insecticide, Fipronil (Lester and Beggs 2019; Edwards *et al.* 2017). We used targeted prediction to identify ligand-gated ion channel (LGIC), olfactory receptor, and spermatogenesis genes using Augustus protein profiles (Keller *et al.* 2011). Our annotation of the Fipronil target site, the GABA receptor *Resistant to dieldrin* (*Rdl*), did not suggest the presence of the classical Ala301 mutation that confers high resistance. *Vespula* LGIC receptors are highly conserved, with one-to-one orthology in *Apis* and *Bombus* (Supplementary Figure 4). This suggests that any chemicals targeting *Vespula* LGICs will also affect bees, as is the case with Fipronil.

## Conclusions

We have produced chromosome-level genome assemblies for three invasive social wasps in the *Vespula* genus. Our approach of short-read sequencing and Hi-C scaffolding using haploid material allowed us to produce assemblies that exceed the genome quality targets suggested by the i5k insect genome sequencing initiative (scaffold *N*_50_ length > 300 kb; Richards and Murali 2015). Using manual curation and computational prediction, we have identified genes that may encode specific biology suitable for targeting with next-generation control technologies, and genes that may be affected by selection by current chemical controls.

These are the first three genomes from this branch of the Aculeata subclade, which will be useful in phylogenetic comparisons of the remarkable life history characteristics of Hymenoptera. In particular, these genomes will be valuable for understanding the evolution of eusociality, which has appeared twice in Vespid wasps independently of other Hymenoptera (Piekarski *et al.* 2018; Hines *et al.* 2007; Peters *et al.* 2017). Comparing the genomes of Vespid wasps, which are highly eusocial, with the closely related paper wasps, which are primitively eusocial, may also help our understanding of how evolution elaborates mechanisms of colonial living.

Vespid wasps are significant invasive pests in many parts of the world. These genomes will be of major importance for applied management of *Vespula*, in programs using both chemical control methods and for next-generation applications. Our assemblies will provide species-specific targets for novel control methods, such as RNA interference, gene drives and the deployment of damaging viruses. The genomic resources we have developed will also be essential for monitoring the effects of next-generation control methods and measuring genomic variation across natural populations.
