# Corrigendum to “High-Quality Assemblies for Three Invasive Social Wasps from the *Vespula* Genus”

**DOI:** 10.1093/g3journal/jkab109

**Published:** 2021-05-03

**Authors:** Thomas W R Harrop, Joseph Guhlin, Gemma M McLaughlin, Elizabeth Permina, Peter Stockwell, Josh Gilligan, Marissa F Le Lec, Monica A M Gruber, Oliver Quinn, Mackenzie Lovegrove, Elizabeth J Duncan, Emily J Remnant, Jens Van Eeckhoven, Brittany Graham, Rosemary A Knapp, Kyle W Langford, Zev Kronenberg, Maximilian O Press, Stephen M Eacker, Erin E Wilson-Rankin, Jessica Purcell, Philip J Lester, Peter K Dearden

*G3*, 10, 3479-3488, 2020. https://doi.org/10.1534/g3.120.401579

In the originally published version of this manuscript, funding information and disclosures were omitted. The following information should have been included after the Acknowledgments section.

## Funding

This project was supported by Genomics Aotearoa (to PKD) and the Biological Heritage National Science Challenge (to PJL) both funded by the Ministry of Business Innovation and Employment (Hīkina Whakatutuki), Government of New Zealand, as well as US National Science Foundation Grant #1655963 and UC Riverside Seed Grant to JP and EWR; Dovetail Genomics Matching Funds Grant to JP.

## Availability of data and materials

Raw reads are hosted in the NCBI Sequence Read Archive under accession PRJNA643352. The version of the assemblies and annotations described in this paper is hosted on Zenodo under DOI 10.5281/zenodo.4001020. The genomes and annotations have also been uploaded to GenBank under accessions JACSDY000000000 (*Vespula pensylvanica*), JACSDZ000000000 (*Vespula germanica*) and JACSEA000000000 (*Vespula vulgaris*). The annotations used in this paper and the annotations hosted by GenBank differ because records were removed to pass NCBI validation.

## Authors’ contributions

PJL and PKD conceived and designed the project. All authors aided in obtaining and analysing the genomic data. PJL, PKD, TWRH, JG, EJR and EJD wrote the manuscript draft, and all authors participated in the revision of the final version. All authors read and approved the final manuscript.

## Ethics approval and consent to participate

Common wasps (*V. vulgaris*) were collected under the permit National Authorisation Number 38337-RES from the Department of Conservation in New Zealand. Samples of other wasps were collected from private land where no permit was required. No other ethical approval was required.

## Competing interests

The authors declare that they have no competing interests.

The above information has now been updated in the online article.

